# Supplementary material for: The Tbx6 Transcription Factor Dorsocross Mediates Dpp Signaling to Regulate Drosophila Thorax Closure
Source: Int J Mol Sci. 2022 Apr 20;23(9):4543. doi: 10.3390/ijms23094543 (PMC9104307; doi:10.3390/ijms23094543)
Supplement: Supplementary file 1 [file ijms-23-04543-s001.zip › ijms-1654453_Supplementary Material.pdf]

The Tbx6 transcription factor Dorsocross mediates Dpp signaling to regulate *Drosophila* thorax closure

Juan Lu[1], Yingjie Wang[1], Xiao Wang[1] , Dan Wang[1*], Gert O. Pflugfelder[2] and Jie Shen[1]*

[1] Department of Plant Biosecurity and MOA Key Laboratory of Surveillance and Management for Plant Quar-antine Pests, College of Plant Protection,China Agricultural University, Beijing 100193, China

[2] Institute of Developmental Biology and Neurobiology, Johannes Gutenberg-University, 55128 Mainz, Germany

*Corresponding author. E-mail: shenjie@cau.edu.cn, dwang@cau.edu.cn, Tel (0086) 10 627 32384

Genetic crossing schemes and resulting genotype:

Fig1D-F: F0*: dpp-Gal4,UAS-GFP/TM6B* X *puc-lacZ/TM6B* → F1: *dpp-Gal4,UAS-GFP/puc-lacZ*

Fig2B: F0: *ap-Gal4,UAS-GFP/CyO* X *UAS-tkv*^{DN}*/TM6B* → F1: *ap-Gal4,UAS-GFP/+; UAS-tkv*^{DN}*/+*

Fig2C: F0: *pnr-Gal4,UAS-GFP/TM6B* X *UAS-pnrRNAi/UAS-pnrRNAi* → F1: *pnr-Gal4,UAS-GFP/ UAS-pnrRNAi*

Fig2D: F0: *pnr-Gal4,UAS-GFP/TM6B* X *UAS-eyg/UAS-eyg* → F1: *UAS-eyg/+; pnr-Gal4,UAS-GFP/+*

Fig2E: F0: *pnr-Gal4,UAS-GFP/TM6B* X *UAS-pnr/CyO-GFP* → F1*: UAS-pnr/+; pnr-Gal4,UAS-GFP/+*

Fig2F: F0: *pnr-Gal4,UAS-GFP/TM6B* X *UAS-tkv*^{DN} */TM6B* → F1: *pnr-Gal4,UAS-GFP/UAS-tkv*^{DN}

Fig2G: F0: *pnr-Gal4,UAS-tkv*^{DN}*/TM6B* X *UAS-pnr/CyO-GFP* → F1*: UAS-pnr/+; pnr-*

Gal4,UAS-tkv^{DN}/+

(Note: pnr-Gal4 and UAS-tkv^{DN} was recombined on the III chromosome for future rescue experiments.)

Fig3B: F0: *pnr-Gal4,UAS-GFP/TM6B* X *UAS-tkv^{DN}/TM6B* → F1: *pnr-Gal4,UAS-GFP/ UAS-tkv^{DN}*

Fig3C: F0: *pnr-Gal4,UAS-GFP/TM6B* X *UAS-pnrRNAi/UAS-pnrRNAi* → F1: *pnr-Gal4,UAS-GFP/ UAS-pnrRNAi*

Fig3D: F0: *pnr-Gal4,UAS-GFP/TM6B* X *UAS-Doc3RNAi/UAS-Doc3RNAi* → F1: *UAS-Doc3RNAi/+; pnr-Gal4,UAS-GFP/+*

Fig3E: F0: *pnr-Gal4,UAS-GFP/TM6B* X *UAS-pnr/CyO* → F1: *UAS-pnr/+; pnr-Gal4,UAS-GFP/+*

Fig3F: F0: *pnr-Gal4,UAS-tkv^{DN}/TM6B* X *UAS-pnr/CyO* → F1: *UAS-pnr/+; pnr-Gal4,UAS-tkv^{DN}/+*

Fig4A: F0: *pnr-Gal4/TM6B* X *UAS-arpc3B-RNAi* → F1: *UAS-arpc3B-RNAi/+* ;*pnr-Gal4/+*

Fig4B: F0: *pnr-Gal4/TM6B* X *UAS-CG12164-RNAi* → F1: *UAS-CG12164-RNAi /+; pnr-Gal4/+*

Fig4C: F0: *pnr-Gal4/TM6B* X *UAS-CG14456-RNAi* → F1: *UAS-CG14456-RNAi /+; pnr-Gal4/+*

Fig4D: F0: *pnr-Gal4/TM6B* X *UAS-CG16758-RNAi* → F1: *UAS- CG16758-RNAi /+; pnr-Gal4/+*

Fig4E: F0: *pnr-Gal4/TM6B* X *UAS*-βtub97EF-RNAi → F1: *UAS*-βtub97EF-RNAi */+; pnr-Gal4/+*

Fig4F: F0: *pnr-Gal4/TM6B* X *UAS*-attc-RNAi → F1: *UAS*-attc-RNAi */+; pnr-Gal4/+*

Fig4G: F0: *pnr-Gal4/TM6B* X *UAS*-mec2-RNAi → F1: *UAS*-mec2-RNAi/+*; pnr-*

Gal4/+

Fig4I: F0: *pnr-Gal4/TM6B* X *UAS*-pcp → F1: *UAS*-pcp/+*; pnr-Gal4/+*

Fig4J: F0: *pnr-Gal4/TM6B* X *UAS*-sdr → F1: *UAS*-sdr/+*; pnr-Gal4/+*

FigS1A: F0: *hs-flp;act>y>Gal4,UAS-GFP/TM6B* X *UAS-dpp/UAS-dpp*→F1: *hs-flp/+;UAS-dpp;act>y>Gal4/+*

FigS1B: F0: *pnr-Gal4,UAS-GFP/TM6B* X *UAS-eygRNAi*→ F1: *pnr-Gal4,UAS-GFP/UAS-eygRNAi*

FigS2A: F0: *pnr-Gal4/TM6B* X *UAS-Doc2-RNAi/UAS-Doc2-RNAi*; *UAS-Doc1-RNAi/UAS-Doc1-RNAi* → F1: *UAS-Doc2-RNAi /+; pnr-Gal4/ UAS-Doc1-RNAi*

Supplementary figures

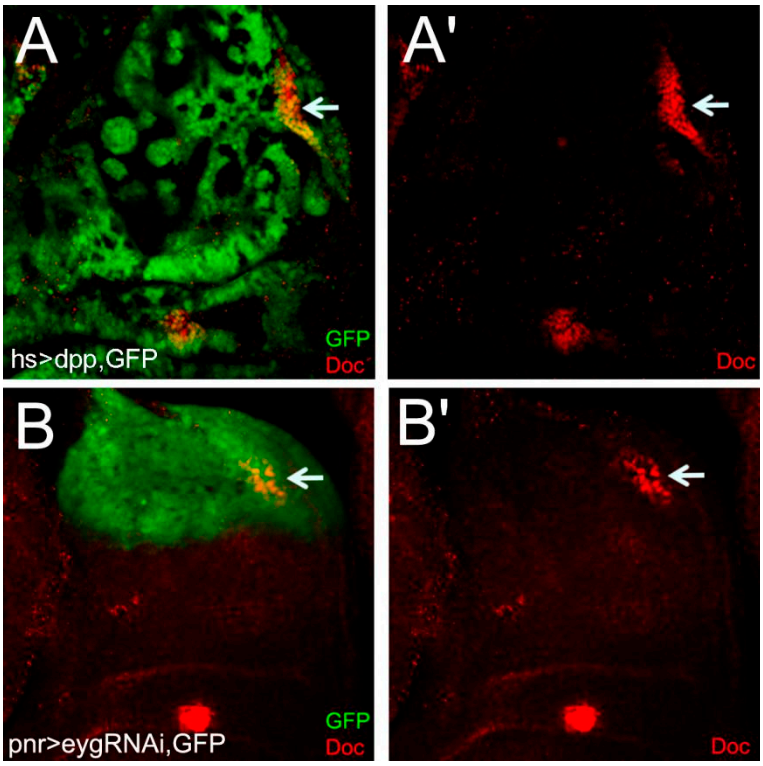

Figure S1 Overexpression of *dpp* (A) or knock-down of *eyg* (B) have no effect on Doc expression in the wing imaginal disc. The white arrow marks the notal Doc expression domain.

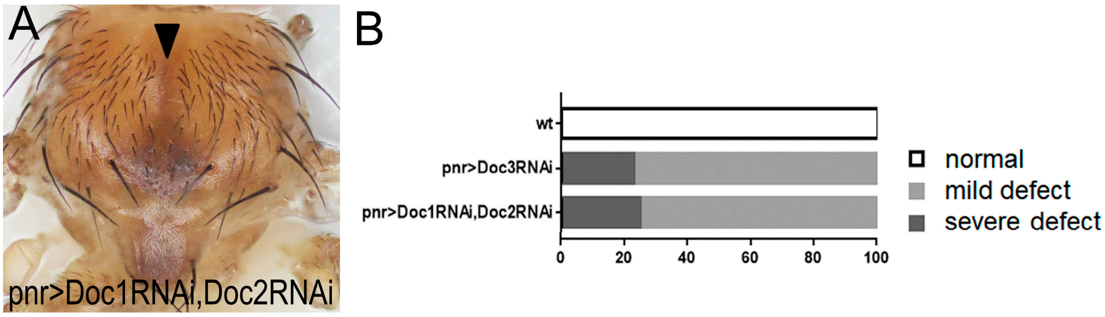

Figure S2 Phenotype of knocking down Doc1+2 and quantification of phenotypes.

(A) Knocking down Doc1+2 induced thorax closure. (B) The phenotype penetrance

between *Doc3-RNAi* expression and *Doc1+2-RNAi* co-expression was similar.

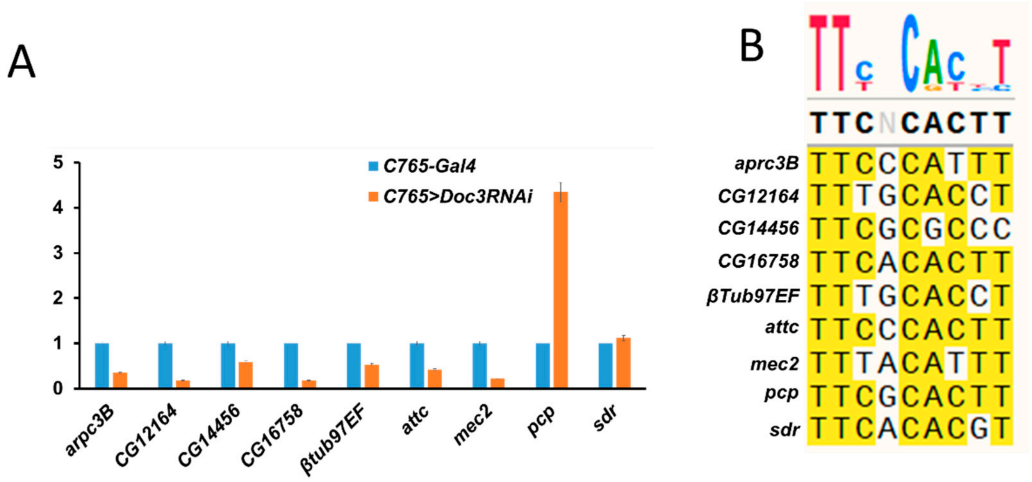

Figure S3 The expression levels and transcriptional binding motif of selected nine genes.

(A) Seven genes (*arpc3B, GC12164, CG14456, CG16758, βtub97EF, attc* and *mec2*) were downregulated in Doc-RNAi while two genes (*pcp* and *sdr)* were upregulated.

(B) The 2 kb upstream region of all nine genes contained a sequence motif related to the Doc binding consensus.
